# Recent Advances in the 3D Printing of Conductive Hydrogels for Sensor Applications: A Review

**DOI:** 10.3390/polym16152131

**Published:** 2024-07-26

**Authors:** Xiaoxu Liang, Minghui Zhang, Cheong-Meng Chong, Danlei Lin, Shiji Chen, Yumiao Zhen, Hongyao Ding, Hai-Jing Zhong

**Affiliations:** 1 Foundation Department, Guangzhou Maritime University, Guangzhou 510725, China; liangxxu@126.com (X.L.); zhangminghui67@gzmtu.edu.cn (M.Z.); 2 State Key Laboratory of Quality Research in Chinese Medicine, Institute of Chinese Medical Sciences, University of Macau, Macao 999078, China; cmchong@um.edu.mo; 3 State Key Laboratory of Bioactive Molecules and Druggability Assessment, Jinan University, Guangzhou 510632, China; carolinelin8@163.com (D.L.); chen94599@163.com (S.C.); zymiu4@163.com (Y.Z.); 4 College of Materials Science and Engineering, Nanjing Tech University, Nanjing 210009, China

**Keywords:** conductive hydrogels, 3D printing, sensors, application, fabrication

## Abstract

Conductive hydrogels, known for their flexibility, biocompatibility, and conductivity, have found extensive applications in fields such as healthcare, environmental monitoring, and soft robotics. Recent advancements in 3D printing technologies have transformed the fabrication of conductive hydrogels, creating new opportunities for sensing applications. This review provides a comprehensive overview of the advancements in the fabrication and application of 3D-printed conductive hydrogel sensors. First, the basic principles and fabrication techniques of conductive hydrogels are briefly reviewed. We then explore various 3D printing methods for conductive hydrogels, discussing their respective strengths and limitations. The review also summarizes the applications of 3D-printed conductive hydrogel-based sensors. In addition, perspectives on 3D-printed conductive hydrogel sensors are highlighted. This review aims to equip researchers and engineers with insights into the current landscape of 3D-printed conductive hydrogel sensors and to inspire future innovations in this promising field.

## 1. Introduction

With the rapid advancement of technology, flexible sensors have been attracting increasing attention. Flexible sensors are a type of sensors with good flexibility and stretchability, which can convert external mechanical stimulation signals (such as pressure, deformation, vibration, etc.) into electrical signal (resistance, capacitance, etc.) output, and realize real-time sensing and monitoring functions [1,2,3,4]. These properties make flexible sensors promising platforms that can be closely bonded to the skin, either directly or indirectly. Therefore, they find wide applications in various fields such as human health testing, electronic skin, implantable devices, soft robots, and motion detection [5,6,7]. Hydrogels, which are three-dimensional hydrophilic bio-inspired polymer networks, can absorb and retain large amounts of water or biological fluids. Their high water content, soft-tissue-like mechanical properties, and biocompatibility make them ideal for flexible sensors, flexible skin, and biomedical and environmental applications [8,9,10,11,12,13,14,15,16,17].

Conductive hydrogels have been extensively developed and emerged as an encouraging platform for the fabrication of wearable electronics, biosensors, tissue engineering, and soft robotics, combining the unique properties of hydrogels, such as softness, biocompatibility, and flexibility, with the electrical conductivity of conductive polymers or composites [2,18,19,20,21,22]. To improve the conductivity of the hydrogel, researchers have incorporated conductive materials like soluble inorganic salts, conductive polymers, carbon-based nanomaterials, and metal nanoparticles into hydrogel matrices [23,24]. When exposed to stress, strain, or electrochemical reaction, the conductivity or resistivity of the hydrogel changes, indicating sensing performance. Therefore, they can be used as electrodes for neural signal recording and stimulation, or as electrochemical sensors for real-time biomarker monitoring [25]. However, the sensor application of conductive hydrogels still faces some challenges. For example, a key challenge is to achieve a balance between mechanical integrity and conductive performance of such conductive hydrogels. The fabrication of hydrogel sensors with low detection limits, high sensitivity, and a wide detection range is still an urgent basic scientific problem to be solved [15]. Establishing a good device/tissue interface between the hydrogel and the soft tissue will help eliminate the interface impedance and improve the signal-to-noise ratio. However, traditional techniques using molds, dies, or masks result in hydrogels with limited structures and shapes. Fabricating conductive hydrogels with tailored structures and properties has been a significant challenge, limiting their practical applications [26,27].

**Figure 1:**
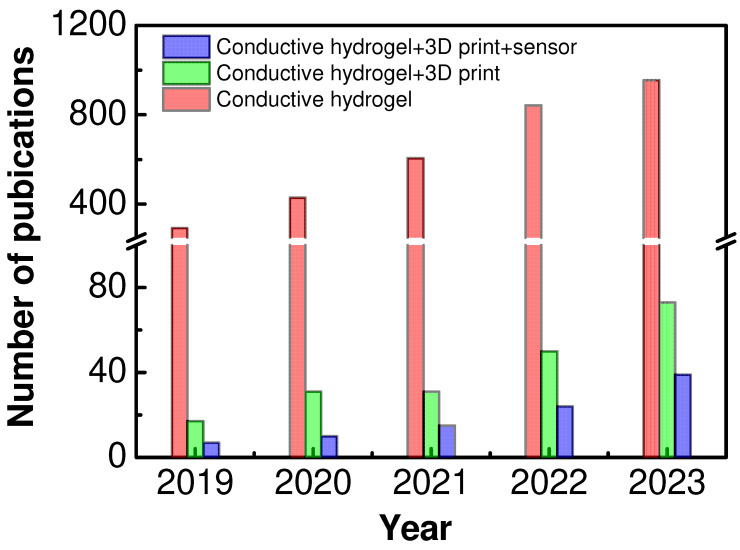
Number of publications over the last five years (from 2019 to 2023) using the keywords “conductive hydrogel”, “3D print”, “sensor”, and their combination, using Web of Science.

In recent years, the progress of 3D printing technology has provided a powerful tool for addressing the challenges associated with the fabrication of conductive hydrogels with tailored structures and properties [2,11]. Unlike traditional preparation methods, 3D printing offers several advantages, including ease of operation, precise structural control, and cost-effectiveness [28,29,30]. The layer-by-layer printing process allows for unprecedented precision and customization in designing and fabricating complex structures. By incorporating conductive materials into hydrogel matrices, researchers have developed numerous innovative strategies for 3D-printing conductive hydrogels with specific properties and geometries. There has also been an increase in the number of relevant publications over the last five years (2019–2023), as shown in Figure 1 [23,31,32,33,34]. This significantly enhances the potential application of conductive hydrogels in flexible sensors and related fields [35,36,37,38,39].

This review article aims to provide a comprehensive overview of the state of the art in the fabrication and application of 3D-printed conductive hydrogel sensors. It covers the fundamental principles, fabrication techniques, and material systems employed in this rapidly evolving field, highlighting the advantages and limitations of each approach. The article delves into the different 3D printing techniques used for conductive hydrogels, including inkjet printing, direct ink writing, stereolithography, and two-photon polymerization, discussing their respective strengths and limitations. Furthermore, the review explores the diverse applications of 3D-printed conductive hydrogel-based sensors, such as human motion detection, strain sensing, temperature sensing, humidity sensing, and gas sensing. Finally, future challenges and perspectives of 3D-printed conductive hydrogel-based sensors are stated. These applications demonstrate the versatility and potential of these materials in addressing real-world challenges in healthcare, environmental monitoring, human–machine interfaces, and soft robotics. It is expected that this review will help researchers gain a thorough understanding of the current state of the field and inspire future innovations in 3D-printed conductive hydrogel sensor technology.

## 2. Conductive Hydrogels

**Figure 2:**
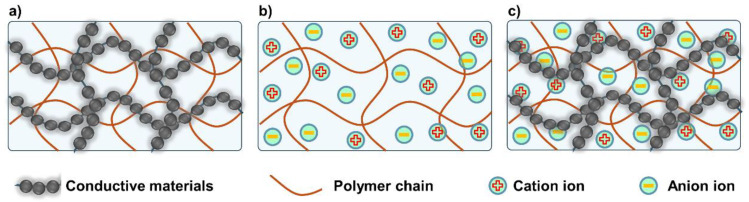
Various types of conductive hydrogels: (**a**) electronic conductive hydrogels (ECH), (**b**) ion conductive hydrogels (ICH), and (**c**) composite conductive hydrogels (CCH).

Hydrogels have a unique porous structure and excellent flexibility, making them highly suitable for applications in flexible electronic skins, wearable electronic devices, and biosensors [12,13,14,15,16]. However, these applications require hydrogels to have good electrical conductivity. Consequently, conductive hydrogel has continuously attracted widespread attention from researchers in recent years [40,41]. Current research on the conductivity mechanism of conductive hydrogels has led to their broad classification into three groups: electronic conductive hydrogels (ECH, Figure 2a), ion conductive hydrogels (ICH, Figure 2b), and composite conductive hydrogels (CCH, Figure 2c) [42,43].

### 2.1. Electronically Conductive Hydrogels (ECHs)

Electronically conductive hydrogels (ECHs) typically consist of two key components: a conductive network and a hydrogel matrix. The conductive network can be formed by incorporating conductive fillers, such as carbon-based nanomaterials or metal nanoparticles, or by in situ polymerization of conductive polymers. The hydrogel matrix can be derived from either insulating or conductive polymers [44,45]. For instance, conductive materials, or conductors, encompass a wide range of materials, including carbon nanotubes, graphene, graphene oxide, metal nanoparticles, and conductive polymers such as polyaniline (PANI), poly(3,4-ethylenedioxythiophene):poly(4-styrenesulfonate) (PEDOT:PSS), and others. These conductive materials can form a three-dimensional network through chemical or physical crosslinking, acting as a hydrogel skeleton and facilitating electron transmission, thereby achieving excellent electrical conductivity [46]. Xu et al. prepared a self-assembled graphene hydrogel (SGH) via a convenient one-step hydrothermal method, which has excellent electrical conductivity as high as 5 × 10^−3^ S/cm and exhibits a high specific capacitance (175 F/g) [47]. Lu et al. developed a pure PEDOT:PSS hydrogel by controlled dry-annealing and rehydration, which exhibits high electrical conductivity (~40 S/cm in deionized water) [48]. However, hydrogels solely composed of these conductive materials often suffer from poor mechanical properties and limited sensitivity, hindering their practical applications [49]. For example, the stretchability of pure PEDOT:PSS hydrogels is relativity poor, with only a 35% elongation at break, and its relative resistance scarcely alters under a 20% tensile strain [48].

On the other hand, the conductive filler is combined into a tough hydrogel matrix polymer network or surface by physical or chemical crosslinking, which can improve the mechanical properties of the conductive hydrogels. However, its performance is often hampered by the inherent non-conductive nature of the hydrogel matrix and the tendency for phase separation between the conductive fillers and the hydrogel network [50]. As a result, this type of ECH typically exhibits relatively low conductivity and sensitivity, limiting its practical applications. Zhang et al. constructed a gradient silver-nanoparticle layer in situ on the polyvinyl alcohol (PVA) hydrogel surface via a green reduction process of Ag^+^ ions. The obtained freezing-thawing-polyvinyl alcohol/Glycyrrhizic acid (FT-PVA/GL) hydrogel is superior to most reported ones in terms of tensile properties (510% and 550 kPa), versatility, and sensitivity (gauge factor, *GF* = 2.84), but the conductivity of the hydrogels is only 6.4 mS/m, which needs further improvement [50].

The conductive polymer precursor solution also can be mixed into the hydrogel precursor solution, and a conductive network that interacts with the hydrogel matrix network can be formed by in situ polymerization, which not only imparts conductive sensing properties to the hydrogel, but also interpenetrates with the hydrogel matrix network to further improve its mechanical properties [46,51]. Liu et al. proposed a novel “doping then gelling” strategy to construct an ultra-stretchable supramolecular polyacrylic-acid/polyaniline (PAA/PANI) hydrogel with an entangled network. The high-density electrostatic interaction between PAA and PANI chains serves as a dynamic bond to initiate the strand entanglement, which enables the PAA/PANI hydrogel with ultra-stretchability (2830%) and high tensile strength (120 kPa). In addition, the PAA/PANI hydrogel-based sensor with high strain sensitivity (*GF* = 12.63), fast response time (222 ms), and robust conductivity-based sensing behavior under cyclic stretching is developed [46]. Peng et al. designed and prepared a novel conductive composite hydrogel via in situ polymerization of conductive polymer PEDOT in PVA aqueous solution and then soaking in polyacrylic acid (PAA) solution and glycerin in two stages. The formation of multiple hydrogen bonds within this composite hydrogel imparted high mechanical strength (~3.6 MPa), and better elongation than pure PVA hydrogels. However, the resultant composite hydrogels exhibited relative low conductivity (~0.95 S/m) [51]. 

### 2.2. Ionic Conductive Hydrogels (ICHs)

Ionic conductive hydrogels (ICHs) consist of physically or chemically doped soluble inorganic salts and ionic liquid (e.g., sodium chloride, lithium chloride, potassium chloride) incorporated into hydrogel matrices such as polyacrylamide (PAM), PAA, and PVA. These hydrogels form a polyelectrolyte network or ion channel, facilitating the directional transfer of free ions and imparting conductivity. Notably, ICHs exhibit low impedance at tissue interfaces and have similar mechanical properties, making them ideal materials for wearable and implantable flexible devices [52,53,54]. For instance, Dong et al. reported an innovative multifunctional hydrogel based on a triple network of sodium alginate (SA), PVA, and PAM with incorporated potassium chloride (KCl) as a conductive additive. The integration of eco-friendly KCl enhances the ionic conductivity and anti-freezing properties of the hydrogel, resulting in impressive performance with a high conductivity of 0.14 S/cm, a *GF* of 6.79 for strain ranges between 380 and 880%, and a *GF* of 11.46 for strain ranges from 880 to 1200%, along with a rapid response time of 268 ms, making it suitable for use in extreme temperature conditions [55]. Zhou et al. developed an innovative poly(acrylic acid-co-1-vinyl-3-butyl imidazole bromide) hydrogel (AAV hydrogel) crosslinked with Al^3+^ ions. The incorporation of ionic liquid segments significantly improved the conductivity of the hydrogel. Compared to previously reported conductive hydrogels crosslinked with Al^3+^ (conductivity of 2.53 S/m), the AAV hydrogel exhibited superior conductivity (12.5 S/m) [56,57].

Inorganic salts can not only provide electrical conductivity, but also adjust the mechanical properties of the polymer network [20,55,58]. Dong et al. found that the exchange of KCl ions with water molecules in SA/PVA/PAM facilitated the ionic crosslinking of PVA and SA [55]. Ding et al. developed a tough and conductive hydrogel formed by incorporating carboxymethyl chitosan and sodium chloride (NaCl) into a polyacrylamide network. The incorporation of NaCl imparted high ionic conductivity (0.52 to 6.44 S/m) to the hydrogel. Furthermore, NaCl played a crucial role in regulating the conformation of the carboxymethyl cellulose (CMC) chains, promoting their extension and formation of more hydrogen bonds with the PAM chains. This synergistic interaction resulted in enhanced mechanical properties, including improved tensile strength, elongation, and toughness, as well as low modulus of the hydrogel [20].

The soft and high-water-content nature of hydrogels poses challenges to the long-term stability of hydrogel bioelectronic applications, with shortcomings such as weak adhesion and dehydration [43]. Zhao et al. developed a new type of ion-conducting hydrogel with improved stability in physiological environments by exploiting the phase separation between polyethylene glycol hydrogels and aqueous salt solutions. These phase-separated aqueous ion solutions create soft, stretchable, and highly ion-conductive circuits within the biocompatible polyethylene glycol hydrogels, preventing unwanted ion diffusion into surrounding tissues. This enhanced stability allows in vivo bioelectronic muscle stimulation through the ion-conducting hydrogel circuits in direct contact with tissue for extended periods of time [10].

Despite the promising properties of ICHs, their high liquid content often results in poor mechanical performance. Conversely, the dense network structure inherent to these hydrogels can impede ion migration, leading to lower conductivity. Typically, ICHs with excellent mechanical properties exhibit lower conductivity, and vice versa, making it difficult to simultaneously attain both desirable traits in a single material system. Achieving an optimal balance between high mechanical strength, toughness, and ion conductivity remains a significant challenge for practical applications of ICHs, which need further improvement [59,60].

### 2.3. Composite Conductive Hydrogels

Aside from the progress stated above, hybrid conductive materials have also been investigated for fabricating conductive hydrogels. Conductive fillers and freely moving ions coexist in a hydrogel matrix. Generally, these resultant hydrogels exhibit better conductivity than individual ECHs and ICHs. For instance, Zhang et al. synthesized a new fully polymeric conductive hydrogel with an interpenetrating polymer network (IPN) structure made of conductive PEDOT:PSS polymers and zwitterionic poly(HEAA-co-SBAA) polymers. The innovative hydrogel demonstrates remarkable properties, including an ultra-high stretchability of 4000–5000%, and a tensile strength of approximately 0.5 MPa, attributed to hydrogen bonding, electrostatic interactions, and IPN structures The integration of zwitterionic poly SBAA and conductive PEDOT:PSS facilitated charge transfer via optimal conductive pathways, endowing the hydrogel with high sensitivity (*GF* = 2.0), enhanced conductivity of 0.625 S/m at low strain, and exceptional nonlinearity under high strains [61]. Zhang et al. fabricated a dual-network ion conductive hydrogel by incorporating polyacrylic acid/graphene oxide–ferric cation/chitosan. The entangled chitosan network grants the PAA/GO–Fe^3+^/CS DN hydrogels a high tensile strength of 2.7 MPa. By immersing the hydrogel, numerous Na^+^ and Cl^−^ ions were introduced, and the hydrogel’s inherent free Fe^3+^ ions provided it with high conductivity, reaching 13.8 S/m [62]. Liu et al. reported hybrid hydrogels formed by interconnecting two networks: a “soft” polymer network of poly(vinyl alcohol) and poly(vinylpyrrolidone), and a “hard” dynamic network of cellulose nanocrystals crosslinked by Fe^3+^ ions. This combined structure offers high strength (2.1 MPa tensile stress), robust toughness (~9.0 MJ/m^3^), reliable stretchability (830% elongation), and good self-recovery. The dynamic Fe^3+^–cellulose bonds dissipate energy under stress, while the soft polymer network ensures smooth stress transfer. The hierarchical porous structure and high water content provide stable, sensitive, and repeatable piezoresistive behavior, making these hydrogels suitable as wearable strain sensors for monitoring human activities and physiological signals [63].

In summary, the electrical conductivity of ECHs generally exceeds that of ICHs due to their unique electronic conducting mechanism [64,65,66]. A synergistic blend of conductive materials and advantageous hydrogel components is essential to balance conductivity, mechanical strength, and sensitivity, paving the way for innovative materials with tailored properties for specific applications.

## 3. 3D Printing Technology for Conductive Hydrogel Fabrication

As mentioned above, traditional methods of preparing conductive hydrogels face the same challenge of coupling the physical and chemical properties of the forming polymer, which affect each other [49,54,67,68]. For instance, modifying the chemical structure to enhance mechanical properties usually leads to the decrease of electrical conductivity [69]. In addition, complex and variable application environments require conductive hydrogels to have diverse structural shapes and high molding accuracy when used as sensors in practical applications. Traditional techniques relying on molds, dies, or masks produce hydrogels with limited structures and shapes, significantly limiting their future application [2,28,70].

Three-D printing is a multifunctional additive manufacturing technique widely used in the preparation of hydrogels with complex structures. Compared with traditional preparation methods, 3D printing offers advantages such as ease of operation, precise structural management, and cost-effectiveness [28,29,30]. By printing layer-by-layer, it is possible to efficiently tailor 3D structural conductive hydrogel-based sensors with diverse functions and structures, as well as high-resolution shapes and sizes [71,72,73,74,75]. However, 3D printing of conductive polymers and their hydrogels faces some difficulties, such as slow reaction kinetics, thermal instability, and phase separation of conductive fillers [28,76,77]. Recent reports provide insight into the successful 3D printing of conductive polymer hydrogels using inkjet printing [78,79,80], direct ink writing (DIW) [34,81,82], digital light processing (DLP) [16,19,83,84], stereolithography (SLA) [85], two-photon polymerization (TPP) [86,87], etc. In this section, we review the recent developments of 3D printing technologies for conductive hydrogels, summarized in Table 1.

### 3.1. Inkjet Printing

Inkjet printing technology has already become a powerful tool in various research fields. Inkjet printing is a 3D printing technique that utilizes low-viscosity fluids ranging from 3.5 to 12 mPa·s. Ink droplets are ejected from a nozzle, only tens of microns in diameter, by a relatively weak driving force, and are precisely deposited onto the print platform, where they solidify through UV curing or heating [79,88]. The ink must possess appropriate flow properties to prevent clogging, substantially limiting the variety of printable materials. The resolution of printed structures varies between 50 and 500 μm. Although inkjet printing effectively creates thin structures on solid substrates, it faces challenges in fabricating self-standing structures [79]. Due to its low viscosity, ink droplets can spread, reducing the resolution and height of printed structures. However, inkjet printing of conductive hydrogels remains highly attractive, thanks to benefits like mask-free operation, relatively high resolution, and support for multimaterial printing [73,78,79,80,89]. For instance, Teo et al. introduced a novel PEDOT:PSS/IL hydrogel using micro-reactive inkjet printing (MRIJP) to pattern various 2D and 3D structures by in-air coalescence of PEDOT:PSS and ionic liquid (IL). By controlling the in-air position and Marangoni-driven encapsulation, single droplets of the PEDOT:PSS/IL hydrogel with diameters as small as about 260 μm are fabricated within about 600 μs. Notably, this MRIJP-based PEDOT:PSS/IL enables free-form patterning while maintaining performance identical to that of materials fabricated using the conventional spin-coating method. This work also marks the first demonstration of inkjet-printed high-concentration alginate hydrogels and free-standing hydrogels on a support without the use of crosslinkers or gel supports [79]. Xie et al. report a facile strategy to construct 3D conductive hydrogels by programmable printing of PEDOT:PSS inside of oil. In this liquid–liquid method, PEDOT:PSS colloidal particles and polydimethylsiloxane surfactants create an elastic film at the liquid–liquid interface, ensnaring the hydrogel precursor inks in specific 3D shapes for later gelation or crosslinking. Conductivities of up to 301 S/m are attained using just 9 mg/mL of PEDOT:PSS in two intertwined hydrogel networks. This approach enables customization of hydrogel elements and characteristics, rendering them ideal for electro-microfluidic devices and specialized NFC implantable biochips [23].

### 3.2. Direct Ink Writing (DIW)

DIW is the most commonly used 3D printing technology, which is known for its high material utilization, diverse ink components (such as nano-conductive particles, nano-conductive fibers, and ionic hydrogels), and comparatively low cost. Through extrusion of viscoelastic precursor solutions and composites, layer-by-layer printing, and subsequent curing, this strategy has proven to be highly versatile for forming hydrogels with complex 3D structures based on digital designs involving both chemical bonding and physical entanglement [34,81,90,91,92]. Unfortunately, a temporary sacrificial material may be needed to support the printed structure since raw viscous materials lack mechanical robustness and can cause collapse of complex shapes. Moreover, hydrogel structures created by nozzle-based printers generally achieve sub-millimeter resolution. Consequently, the incorporation of these structures into capacitive sensors severely restricts their capacity to improve sensor sensitivity [29,71,93,94].

For instance, Zhou et al. developed a bi-continuous conducting polymer hydrogel (BC-CPH) that simultaneously achieves high electrical conductivity (11 S/cm), stretchability (over 400% strain), and fracture toughness (over 3300 J/m^2^) in physiological environments. The bi-continuous structure, formed by the phase separation of the electrical (PEDOT:PSS) and mechanical (polyurethane) phases, overcomes the traditional trade-off between conductivity and mechanical robustness in conducting polymer hydrogels. This advancement makes BC-CPH readily applicable to advanced fabrication methods, including DIW. This material innovation provides a versatile platform for hydrogel bioelectronics and conducting polymer applications [92]. Liu et al. proposed a hierarchical fabrication strategy for creating strong and tough ceramic-reinforced organo-hydrogels. During fabrication, the hydrogel matrix is substituted in an ionic solution containing FeCl_3_, allowing the incorporation of Fe^3^⁺ and Cl⁻ ions. Individual filaments containing aligned platelets are DIW 3D-printed into bio-inspired macro-architectures, resulting in high stiffness, strength, and toughness (fracture energy up to 31.1 kJ/m^2^) achieved through multi-scale energy dissipation mechanisms. The incorporation of Fe^3^⁺ and Cl⁻ ions also imparts high electrical conductivity, up to 8.8 S/m. This study demonstrates the extension of natural design principles to fabricate composite hydrogels with synergistically enhanced mechanical and functional properties [95].

### 3.3. Digital Light Processing (DLP) and Stereolithography (SLA) 

Photopolymerization is one of the most advanced 3D printing technologies, mainly including SLA and DLP processes, and light-curing hydrogels can be used [83,96]. During fabricating, a photosensitive polymer initiates polymerization when exposed to ultraviolet (UV) light. The polymer photopolymerizes at specific locations within the same layer to create the desired pattern, and the final 3D structure is obtained by successive layer-by-layer illumination [19,83,85]. Specifically, SLA uses a laser source to write directly onto the polymer surface, to be able to create free-form, complex 3D architectures; DLP uses digital light projection to cure the entire pattern of the layer simultaneously. Both SLA and DLP are widely used to fabricate flexible sensors with complex geometries [96,97,98]. However, light-cured hydrogels are generally more expensive, exhibit lower strength, and are prone to shrinkage during the polymerization process, calling for further improvements [19,83,85]. 

For example, Dong et al. described a 3D-printable hydrogel merging biocompatibility and structural integrity with high electrical conductivity from PEDOT:PSS, via SLA 3D printing. These structures offer strong support for delivering electrical stimulation to encapsulated cells such as dorsal root ganglion (DRG) neurons. This novel conductive 3D-printable hydrogel enables precise patterning of conductive structures that interface with biological systems for electrical stimulation and signal recording applications [85]. Robert et al. developed an SLA strategy to create architected conducting polymer hydrogels with intricate lattice structures. By utilizing architectural design principles, they effectively decoupled the mechanical and electrical properties of these hydrogels from their intrinsic chemical composition. This approach allows for the tuning of mechanical properties such as elasticity, fracture strain, and damage tolerance through the 3D geometry of the lattices, rather than being constrained by the materials’ chemical makeup. Consequently, the architectural design enables rational adjustments of the typically interdependent mechanical and electrical properties, addressing the limitations of traditional composition-based approaches for conducting polymer hydrogels. The unique combination of elasticity, pliability, stable electrical properties, and damage tolerance enables use in emerging dynamic applications like soft robotics, wearable electronics, etc. [98]. 

On the other hand, DLP offers high resolution and fast print speeds. Most importantly, DLP does not require a highly transparent resin, allowing for greater flexibility in material selection [99]. For instance, Yin et al. fabricated a new hydrogel formed by co-polymerizing two photo-curable hydrogels with varied mechanical properties: flexible PAAm and tougher PEGDA. The addition of MgCl_2_ improved ionic conductivity and water retention of the obtained hydrogels. This combination allows for tunable mechanical properties, achieving high stretchability and elasticity similar to human skin. Using a commercial DLP 3D printer, they printed ionically conductive hydrogels into complex microstructures with high resolution (down to 150 μm). This innovation enables the easy fabrication of highly sensitivity wearable sensors, advancing smart electronics and artificial intelligence applications [71]. He et al. developed a novel hydrogel structure via DLP printing. By combining an aqueous polyurethane microemulsion with a standard hydrophobic photo-initiator and employing a dual-material strategy to control dehydration, the obtained hydrogels exhibited excellent strength (high strength of 22.9 MPa, elasticity of 583%) and ionic conductivity (up to 9.64 S/m) through chemical crosslinking and ion coordination. Using dual-material 3D printing, the hydrogel was packaged with elastomers. These hydrogel-based sensors, indicating high sensitivity and reliability under stretching and compressive deformation, were applied to monitor various human motions [84].

### 3.4. Two-Photon Polymerization (TPP)

Two-Photon Polymerization (TPP) relies on the simultaneous absorption of two photons in the photopolymer to cure features with submicron resolution. This allows local polymerization of the photosensitive material and the fabrication of complex 3D structures with nanoscale precision [86,87,100]. Beyond commercially available acrylate resins, researchers have explored multifunctional polymers with fillers or hydrogels to expand the applications of TPP technology. Notably, TPP has fewer restrictions on the rheological properties like viscosity of the hydrogel material compared to methods like direct ink writing or inkjet printing that have stringent rheology requirements [70,101]. As a result, TPP has been successfully used to print functional devices for various applications, including flexible electronics, hierarchical structures, and 3D scaffolds [31,102]. For example, Paola Sanjuan-Alberte et al. presented a novel TPP strategy to fabricate micro/nanoscale structures using gelatin methacrylate (GelMa) hydrogels with enhanced electrical properties. By dispersing multi-walled carbon nanotubes (MWCNTs) as conductive nanofillers, the electrical properties were significantly improved, showing lower impedance values and improved ability to exchange electrons compared to pristine GelMa. This material supports the viability and growth of human-induced pluripotent stem-cell-derived cardiomyocytes (hPSC-CMs). Ultra-thin film structures (10 µm thickness) and scaffolds were successfully fabricated, demonstrating the potential of this method for tissue engineering and bioelectronics [87]. Lichade et al. reported a photosensitive PEDOT:PSS-PEO-based conductive hydrogel (PPCH) and its direct patterning via a two-photon polymerization (TPP) process. The optimal PPCH composition (10 wt% PEO) and TPP printing window were determined to achieve high-resolution (down to 500 nm) and stable 3D microstructures. The printed PPCH microdevices demonstrated functionality for micro-energy storage (capacitors), biosensing (alcohol sensing), and tissue engineering (supporting stem cell growth) due to their conductivity and biocompatibility [86].

However, TPP still has limitations, particularly when applied to hydrogels. For example, not all hydrogels are compatible with TPP, as it requires photo-initiators that can absorb two photons simultaneously, restricting the range of usable materials. Additionally, some photo-initiators employed in TPP can be toxic, presenting challenges for biomedical applications [31,70,101,102].

## 4. Application of 3D-Printed Conductive Hydrogel Sensor

Conductive hydrogels combine the electrochemical properties of conductive polymers with the softness and biocompatibility of hydrogels, making them highly valuable for wearable electronic devices, flexible sensors, and tissue engineering [4,5,6]. The unique advantages of 3D printing technology, including material versatility, convenience, customizability, environmental friendliness, etc., can effectively address the current challenges of conductive hydrogels, including low conductivity, limited sensing sensitivity, and restricted material shapes and structures. This broadens the application potential of conductive hydrogels in flexible sensors and related fields [19,43,85,103,104,105,106,107,108]. In this section, we review the recent developments in 3D-printing conductive hydrogel sensors, summarized in Table 2.

### 4.1. Human Motion Detection Sensors

**Figure 3:**
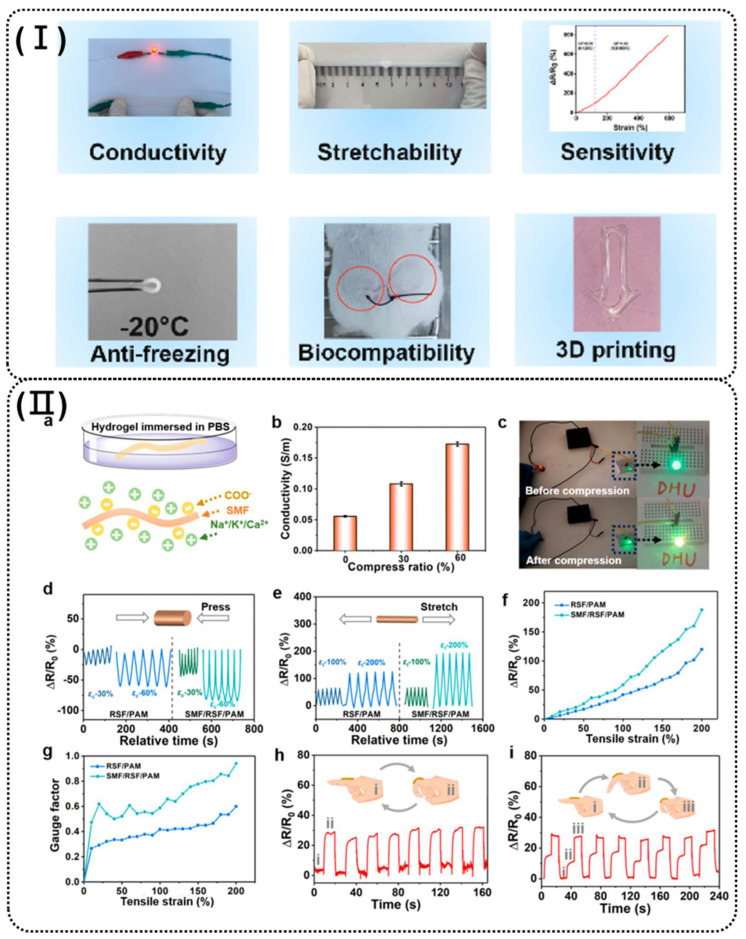
(**I**) Schematic diagram of properties of PPNGC hydrogel [111]. (**II**) Conductivity and strain-sensing ability of 3D-printable triple-network composite hydrogels. (**a**) Hydrogel is immersed in PBS (10 mM), and the Na+/K+/Ca2+ ions are attracted by SMF. (**b**) Conductivity of the SMF/RSF/PAM hydrogel-based strain sensor with different compression ratios. (**c**) Photographs of the LED light before and after compression. Strain sensors of hydrogels under (**d**) compression and (**e**) tensile loading. (**f**) Relative resistance changes and (**g**) GF variations of the composite hydrogel-based sensors under the applied tension. Relative resistance changes upon (**h**) two and (**i**) three movements of the strain sensor on the fingers. [112].

Hydrogel-based sensors serve as an ideal platform for developing personalized wearable electronics in real-time healthcare and motion detection due to their high flexibility, conformability, and sensing performance [53,109,110]. In addition, advances in 3D printing technology provide the opportunity to fabricate various sensors from conductive hydrogels. This is possible because of the low processing costs, superior fabrication accuracy, and satisfactory production efficiency provided by 3D printing [4,32,70,74,108]. For instance, Jiang et al. developed a 3D-printable hydrogel using a freeze-thaw method with polyvinyl alcohol, polyvinylpyrrolidone, sodium chloride, glycerol, and sodium carboxymethyl cellulose (PPNGC hydrogel, as shown in Figure 3I). This hydrogel demonstrated excellent electrical conductivity (2.1 S/m) and mechanical properties within the temperature range of −40 °C to 20 °C. It also showed high sensitivity (*GF* = 0.78 in the strain range of 0–120% and 1.52 in the strain range of 120–600%) and cyclic stability, effectively monitoring both large-scale human motions (wrist, elbow, knee flexions) and subtle movements in real time [111]. Sun et al. fabricated an eco-friendly biocompatible hydrogel sensor based on PANI and gelatin via a 3D printing method. The sensor exhibited a stress sensing range of 0–899.8 MPa, a strain sensing range of 0–764.4 % sensitivity (*GF* = 1.4), and a TCR = −1.3 for temperature 8.4–29 °C. The sensor was also capable of accurately detecting both large strains in human knee joints and small strains in finger bending [108]. Niu et al. presented a novel 3D-printable triple-network hydrogel composed of silk microfibers (SMF), regenerated silk fibroin (RSF), and PAM, with enhanced mechanical properties like high elastic modulus, compressive strength, and stretchability (SMF/RSF/PAM composite hydrogel, as shown in Figure 3II). The negatively charged SMFs enable the hydrogel to adsorb cations from solutions such as phosphate-buffered saline (PBS), providing good ionic conductivity and strain-sensing capability (*GF* = 0.95) even at low salt concentrations. This hydrogel can accurately detect different degrees of finger bending by monitoring resistance changes [112].

**Figure 4:**
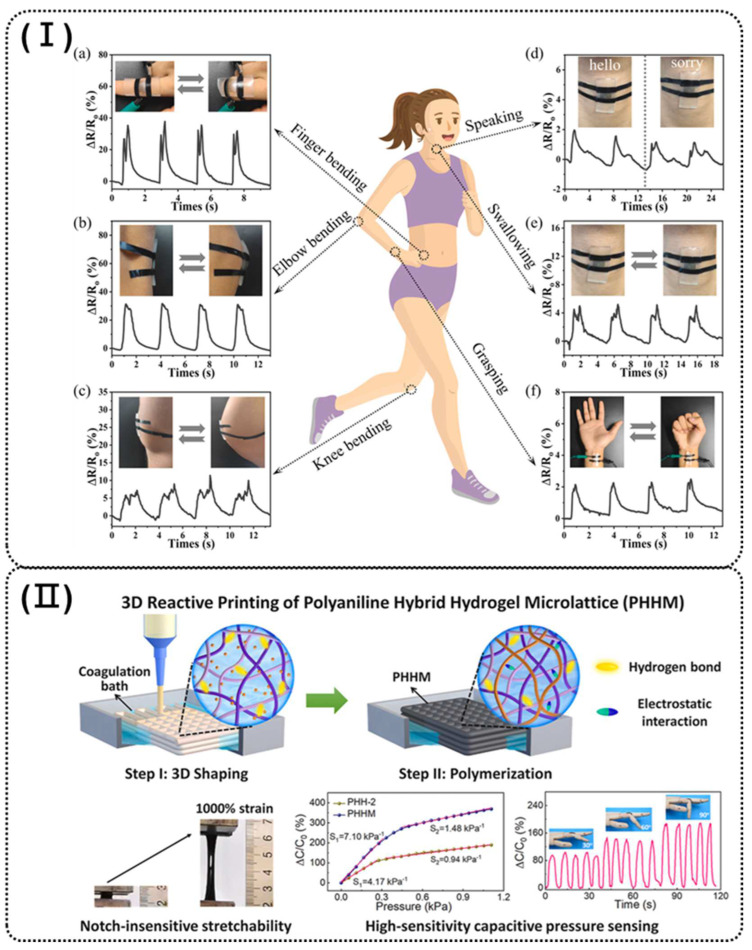
(**I**) Digital photographs and corresponding relative resistance change of the poly(ACMO)/Pt sensor attached to the (**a**) finger, (**b**) elbow, (**c**) knee, (**d**,**e**) throat, and (**f**) wrist [74]. (**II**) Schematic of the preparation procedure and properties of 3D reactive printing of the PHH hydrogels (polyaniline hybrid hydrogels) [32].

To improve the dispersion stability of conductive fillers in hydrogels, Guo et al. developed an electrically conductive and degradable hydrogel-based strain sensor using digital light processing (DLP) technology (as shown in Figure 4I). By using an ion-sputtering strategy, they avoided complex modifications and achieved an electrical conductivity of 1.6 S/cm. The poly N-acryloylmorpholine/Pt hydrogel (poly(ACMO)/Pt hydrogel) sensor demonstrated a *GF* of 1.5 at 0–10% strain and 7.2 at 10–100% strain, along with good cyclic stability and suitability for monitoring various human activities. This study highlights the potential of 3D-printed conductive hydrogels for eco-friendly strain sensors and next-generation flexible electronics [74].

Conductive hydrogels typically struggle to achieve a balance between high elasticity, sensitivity, and a wide response range for flexible pressure sensing [32,53]. To address this issue, Yue et al. introduced a reactive shaping method to prepare polyaniline hybrid (PHH) hydrogels with dual electron/ion conductivity (as shown in Figure 4II). These hydrogels feature reversible hydrogen bonds and electrostatic interactions that act as sacrificial bonds, giving them remarkable stretchability (>1500%) and high fatigue resistance (maintaining >80% of maximum stress and elastic modulus after 2000 cycles). Additionally, this method allows for 3D reactive printing of PHH with high elasticity and dual conductivity, rendering them appropriate as stretchable conductors for capacitive pressure sensors of high sensitivity (7.10 kPa^−1^) across a broad sensing range. This method introduces new opportunities for 3D-printable flexible sensors, with potential uses in electronic skin prosthetics, human–machine interfaces, and physiological signal detection [32]. In general, 3D-printable tough hydrogels face challenges in replicating complex biological structures due to their relatively weak mechanical properties and uniform (isotropic) structures, making it difficult to accurately mimic living tissues such as tendons. As a result, these hydrogels are not yet suitable for applications that require significant load-bearing capacity [36]. To address this issue, Yue et al. enhance 3D-printed hydrogels with anisotropy and exceptional mechanical properties through a simple stretching-fixing-equilibrating process. By adjusting the pre-stretch ratio and SA content, the hydrogel exhibited outstanding mechanical properties (tensile strength of 9–44 MPa; elongation at break of 120–668%; Young’s modulus of 7–62 MPa; toughness of 26–52 MJ m^−3^). Additionally, the incorporation of small amounts of SA plays a key role by inducing strong intermolecular interactions and increasing ink viscoelasticity, which stabilizes anisotropy and improves shape fidelity. This innovative approach enables the creation of tough, anisotropic hydrogels with complex structures, making them suitable for load-bearing components and self-powered wearable electronics in human motion detection [36].

### 4.2. Healthcare Detection Sensors

Bioelectronic devices facilitate efficient communication between medical instruments and human tissue, directly addressing various neurological disorders [48]. Hydrogel-based electronic devices show great promise for recording biological signals and stimulating tissue because of their similarity in mechanical properties to human tissue. Hydrogels provide a highly hydrated and biocompatible environment that mimics biological tissues. Their conductive components enable electrical stimulation and sensing. The unique characteristics of hydrogels create opportunities for advancements in bioelectronics, biosensors, electrically controlled drug release, and tissue engineering. Additionally, the use of 3D printing to create intricate, complex shapes of conductive hydrogels facilitates the advancement of advanced bioelectronic tools and interfaces for medical purposes [19,43,85,103,104].

In Dong and his team’s study, the 3D-printable conductive dorsal root ganglion (DRG) cell-encapsulated gelatin methacryloyl (GelMA) hydrogels containing PEDOT:PSS were synthesized. The hydrogels demonstrated excellent biocompatibility, supporting the adhesion and proliferation of dorsal root ganglion (DRG) neural cells. The 3D-printed hydrogel structures efficiently transferred electrical stimulation to the encapsulated DRG cells, enhancing their neuronal differentiation and the expression of neurogenic markers such as BDNF, NT-3, and erbB2. This system provides an effective platform for neural tissue engineering by integrating structural support, electrical conductivity, and the capability to deliver electrical cues to modulate cell behavior [85].

In a novel approach, Fantino et al. integrated the 3D printing of hydrogel structures with a subsequent interfacial polymerization step to incorporate a conductive polymer, polypyrrole (PPy), within the hydrogel matrix (PEGDA). This method allows for the creation of intricate, well-defined three-dimensional shapes of conductive hydrogels with precise control over microstructure and conductivity. By combining precise 3D printing with the conductivity of PPy, conductive hydrogels with outstanding electrical properties (low resistivity) are produced, while also preserving sufficient mechanical properties. Compared to directly blending conductive fillers into hydrogel precursors before 3D printing, this method provides greater precision. It facilitates the production of advanced bioelectronic interfaces and devices for various applications, including biosensors, drug delivery, and tissue engineering [19].

**Figure 5:**
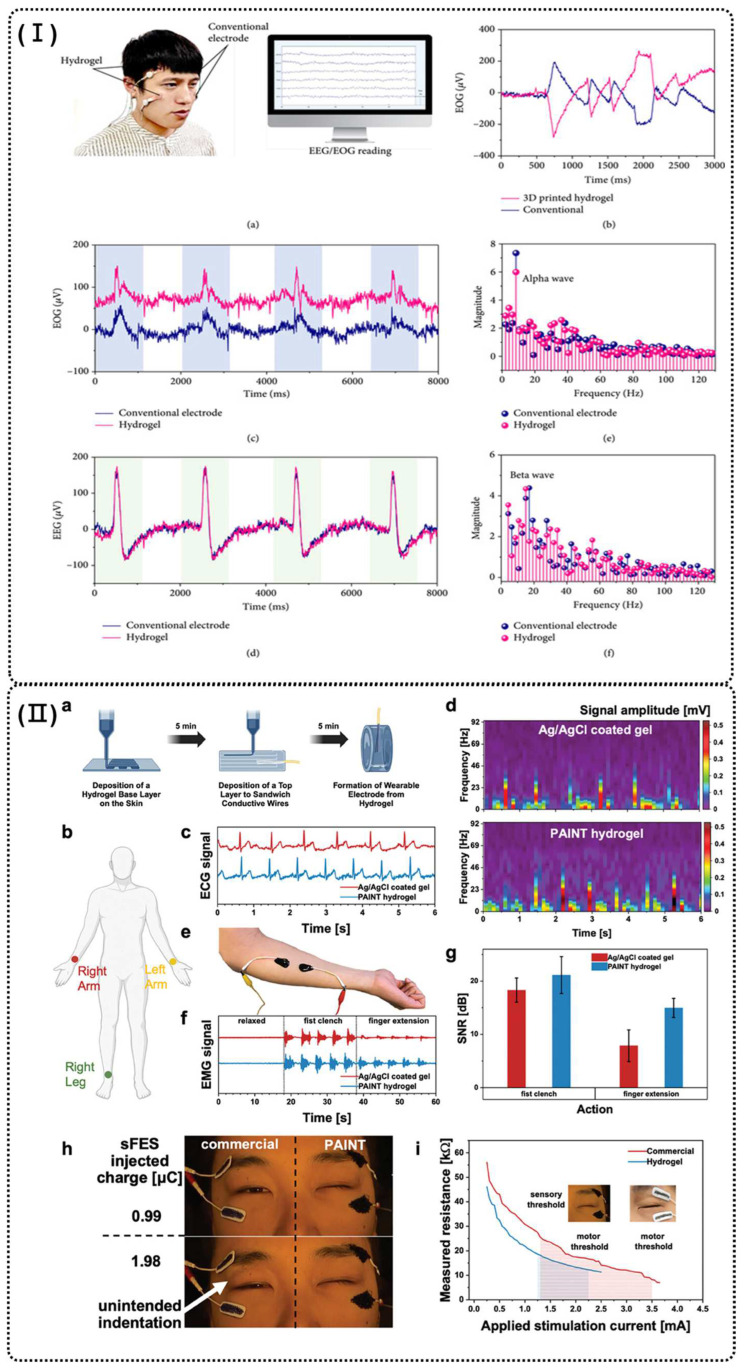
(**I**) Performance of a flexible electrode made of the printed hydrogel for capturing human neural signals. (**a**) Illustration of the 3D printed flexible electrode acting as a human-machine interface. (**b**) The EOG of the nerve of horizontal rotation of the eye balls: the opposite signals indicate the EOG of right and left eyeballs. (**c**) The EOG signal of blinking the eyes. (**d**) The EEG signal of blinking the eyes. (**e**) The Fourier transformation of the signal of closing the eyes and relaxing. (**f**) The Fourier transformation of the signal of opening the eyes and focusing to show the neural activity [106]. (**II**) (**a**) Schematic illustration of the manufacturing process of epidermal PAINT hydrogel electrodes with direct ink writing. (**b**) Schematic of electrode setup for ECG tests. (**c**) Biopotential ECG signals monitored with Ag/AgCl coated gel and PAINT hydrogel electrodes of identical surface area. (**d**) Frequency-amplitude spectrogram of the recorded ECG. (**e**) Schematic of electrode setup for sEMG tests. (**f**) sEMG of fist motions monitored with Ag/AgCl coated gel and PAINT hydrogel electrodes of identical surface area. (**g**) Signal-to-noise ratios show a 16.7% improvement for the fist clenching sEMG and an 87.5% improvement for the finger extension sEMG with the PAINT hydrogel. (**h**) Comparison between commercial and PAINT electrodes during eye closure sFES. (**i**) Resistance as a function of stimulation current during sFES of the eye closure [2].

The electroencephalogram (EEG) and the electrooculogram (EOG) are very important probes of neural signals in humans. Wang et al. fabricated 3D-printed hydrogels (as shown in Figure 5I), made of PAM, PEGDMA, LiCl, that offer high stretchability (2500%), conductivity, and ultra-low working voltage (<100 μV) and freezing point (−125 °C). The present hydrogels show potential as flexible electrodes for acquiring human electrophysiological signals (EOG and EEG), enabling accurate recording of alpha and beta brain waves. This highlights their promise for human–machine interface development, particularly for next-gen smart electronics in extremely low-temperature conditions [106]. Chen et al. reported a 3D-printable bio-sourced conductive hydrogel (PAINT, as shown in Figure 5II) of CMC, polydopamine (PDA), and myoinositol hexakisphosphate or phytic acid (PA), as well as the conductive polymer PEDOT:PSS, due to their biocompatibility and physical crosslinking. This hydrogel, used for ECG and surface electromyography (sEMG) signal collection, also served as surface functional electrical stimulation (sFES) electrodes, delivering 40 Hz AC during facial sFES of the orbicularis oculi. The in situ-formed hydrogel electrodes achieved an 88% improvement in signal-to-noise ratio for forearm muscle biopotential monitoring and reduced the required current from 3.5 to 2.25 mA for functional electrical stimulation of eye closure. These results demonstrated its potential for future healthcare applications with enhanced personalization, efficiency, and comfort [2]. On the other hand, long-term stability is essential not only for conductive hydrogels in practical applications, but also for their precursor inks. Yu et al. developed a 3D-printable conducting polymer hydrogel (CPH) for electrical bioadhesive interfaces (EBI), composed of PEDOT:PSS, poly sulfobetaine methacrylate (PSBMA), and PVA. The inks remained stable for 1 month without aggregation, thanks to molecular interactions like chain entanglement and hydrogen bonding. The PEDOT:PSS-based EBI also showed excellent electrochemical stability, with only a 12.27% reduction in charge injection capacity after 10 million cycles. These features make PEDOT:PSS-based EBIs promising for biomedical applications, including health monitoring and electrophysiological diagnostics [9].

In summary, the advancement of hydrogel-based bioelectronic devices represents a significant leap forward in the development of medical devices and interfaces. These devices, due to their mechanical similarity to human tissue and their conductive properties, create a highly compatible and functional platform for biological signal recording and tissue stimulation. The integration of 3D printing technology further enhances these capabilities by enabling the creation of complex and intricate structures that are essential for advanced biomedical applications.

### 4.3. Environmental Detection Sensor

3D-printed conductive hydrogels have also emerged as promising materials for environmental detection applications due to their unique properties, including high sensitivity, flexibility, and biocompatibility. They can be customized to react to different environmental stimuli like humidity, temperature, pH, and light, making them versatile sensors for tracking and responding to environmental changes [38,113,114,115,116,117].

#### 4.3.1. Humidity Sensors

Humidity is another important and common variable that greatly affects our health and daily comfort. Humidity sensors are widely used in environmental monitoring, healthcare, and the design of electronic and optical devices. Monitoring, detection, and control of ambient humidity are critical in many manufacturing processes [118,119]. Despite numerous advances in the development of humidity sensors, the field still faces significant technical challenges related to material design and structural engineering [41,115,116,120,121]. Due to its intrinsic stretchability, the conductive hydrogel can conform to complex surfaces and withstand deformations, rendering it an optimal material for wearable and flexible humidity-sensing applications. The high conductivity and sensitivity of these hydrogels permit precise measurement of humidity, which is crucial for applications where small changes in moisture levels must be detected and responded to promptly [113,115,122]. Furthermore, the versatility and customization offered by 3D printing techniques have the potential to significantly enhance the performance, integration, and applicability of conductive hydrogel-based humidity sensors [113,114,115,116].

**Figure 6:**
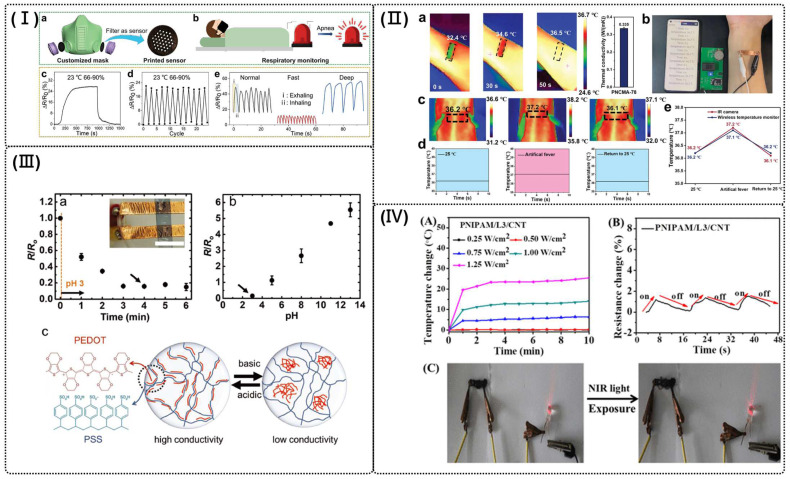
(**I**) Humidity sensitivity and potential applications of the graphene nanoplate–carbon nanotube (GC) humidity sensor. (**a**) A filter with sensor function and (**b**) its application scenarios; (**c**) The resistance changes of the GC sensor from the indoor environment to the high humidity environment and back to the indoor environment; (**d**) Cyclic testing of the GC humidity sensor; (**e**) Respiration testing results of normal breath, fast breath, and deep breath [115]. (**II**) Wireless temperature monitor based on PNCMA-78 deep eutectic gel. (**a**) Thermal conductivity of PNCMA-78 gel. (**b**) Photo of the wireless body temperature monitor, while the meaning of the non-English term is Bluetooth networking. (**c**) Infrared images captured body surface temperature at 25 °C, artificial heating, and returning to 25 °C. (**d**) Temperature-time curves drawn by the data collected on the mobile phone. (**e**) Temperature change curves in three states by using a deep eutectic gel sensor and an infrared camera, respectively [117]. (**III**) pH response of PEDOT:PSS/hydrophilic polyurethanes (HPU) hydrogel-based sensor. (**a**) an example of the electrical response of PEDOT:PSS/HPU hydrogels to the pH change from neutral to pH 3 over time with inset showing an image of the sample with electrical connections and (**b**) correlation between resistance and pH of the solution for PEDOT:PSS/HPU hydrogels. (**c**) Schematic illustration of the impact of pH on molecular structure of PEDOT:PSS [123]. (**IV**) Temperature changes of poly (N-isopropyl acrylamide) (PNIPAAM) /L3/ carbon nanotubes (CNTs) hydrogels (**A**) after NIR light exposure. Relative resistance changes of PNIPAM/L3/CNT hydrogels under NIR light exposure (**B**). Photographs of the increased light intensity after NIR light exposure (**C**) [38].

Hou et al. developed a novel conductive ink combining graphene nanoplates (GNPs) and carbon nanotubes (CNTs). The ink was subsequently utilized for multi-jet fusion (MJF) printing to fabricate strain and humidity sensors aimed at predicting human motion and monitoring health (as shown in Figure 6I). The ink demonstrated excellent dispersibility, effectively preventing the aggregation of GNPs. Additionally, it exhibited commendable conductivity, measured at 3.18 S/m, facilitating the accurate sensing of resistance variations in response to strain and humidity. This innovative conductive hydrogel presents new opportunities for healthcare applications, including human motion prediction, breath mode monitoring, and vital sign detection [115].

Li et al. demonstrates the 3D printing of composite hydrogels made of poly(ethylene glycol) diacrylate (PEGDA) and multi-walled carbon nanotubes (MWCNTs) using a multimaterial mask image projection stereolithography process. The PEGDA/MWCNT composite shows an increase in resistance due to the swelling-induced increase in distance between MWCNTs, while resistance increased from 770 kΩ to 17.25 MΩ upon absorption of 8 μL water (22× increase). In addition, the 3D printing approach enables fabricating sensors with high resolution (40 μm) and customized complex geometries not possible with traditional manufacturing methods. The 3D-printed liquid sensor offers us a unique perspective on the potential applications in various fields for detecting liquid leakage with precise position and direction [116].

#### 4.3.2. Temperature Sensor

Beyond humidity sensing, such 3D-printed conductivity hydrogels can be engineered to detect other parameters like strain, temperature, or pH, leading to multifunctional sensing platforms that can provide comprehensive environmental monitoring [33,115,116]. 

It is essential and of great significance to impart high mechanical performance, environmental stability, and high sensitivity to emerging flexible temperature sensors. For instance, Choi et al. introduced a simple fabrication method using 3D-printed molds to create customized patterns of liquid metal electrodes encapsulated within a hydrogel matrix. This setup integrated three sensor units: a photoresistor, a thermistor, and a tilt switch. Each sensor displayed distinct and reasonable changes in electrical resistance in response to light, heat/cold, and tilting, respectively. Initially, sensors were tested individually with their specific stimuli and showed clear responses. Subsequently, the sensors were combined into a multimodal system that successfully detected and discriminated among multiple simultaneous stimuli [33]. Li et al. prepared poly (N-isopropyl acrylamide) (PNIPAAM) gel using 3D printing, with a conductive PANI polymer network in situ-polymerized on the PNIPAAM matrix. These hybrid hydrogels possess excellent tensile properties, temperature sensitivity, and electrical conductivity. They exhibit rapid and significant volume changes in response to temperature variations around the PNIPAAM component’s lower critical solution temperature (LCST) of 35 °C. The swelling and deswelling of the PNIPAAM matrix shift the conductive PANI network, altering electrical conductivity with temperature changes, making them ideal for thermally responsive applications [124]. In Yao et al.’s work, polymerizable deep eutectic solvents were created by mixing N-cyanomethyl acrylamide (NCMA) with lithium bis(trifluoromethane) sulfonimide (LiTFSI), resulting in supramolecular deep eutectic polyNCMA/LiTFSI gels post-polymerization. These gels exhibit excellent mechanical properties (tensile strength of 12.9 MPa and fracture energy of 45.3 kJ/m^2^) and high-temperature responsiveness, along with good environmental stability and 3D printability. As a flexible temperature sensor, the polyNCMA/LiTFSI gel-based wireless monitor demonstrated high thermal sensitivity (8.4%/K) over a wide range and has potential use as a pressure sensor (as shown in Figure 6II) [117].

#### 4.3.3. pH Sensor

pH is also a crucial environmental indicator that offers valuable insights into the surrounding environment. Monitoring pH is vital for maintaining control and stability in different applications to guarantee safety, efficacy, and efficiency. It underpins advancements in healthcare, environmental protection, industrial production, agricultural practices, and scientific research [125,126,127]. Recently, novel pH sensors utilizing 3D-printed conductive hydrogels have been created and employed to accurately measure pH levels [107,123]. For instance, Naficy et al. created a 3D-printed hydrogel composite by combining pH-sensitive, conductive PEDOT:PSS with a tough, flexible hydrophilic polyurethane (HPU) hydrogel matrix. This composite is mechanically robust, electrochemically active, and responsive to pH changes, showing a linear resistance ratio from pH 3 to 13, which was attributed to the ionic interactions between the PEDOT and PSS components, where the distribution and doping levels of PEDOT along the PSS chains changes with pH (as shown in Figure 6III). The sensors remained stable for over two months in water and pH buffer solutions, making them suitable for use as flexible pH sensors in wet environments and biological applications [123]. Rocco Carcione et al. created flexible 3D-printed polyethylene glycol diacrylate (PEGDA)-sulfonated polyaniline (PANI) electrically conductive hydrogels (ECHs) for pH-monitoring applications. The PEGDA–PANIs hydrogel was successfully tested as an electrode material for pH monitoring, providing a linear detection response within the pH range of 2–7, which makes it a promising active material for developing advanced biomedical, soft-tissue, and biocompatible electronic applications [107].

#### 4.3.4. Light Sensor

Upon exposure to near-infrared (NIR) light, the conductive hydrogels (composed of CNTs, graphene, ppy, and polyaniline) efficiently absorbed and converted it into heat, demonstrating photothermal properties [38,128]. For example, Deng et al. presented multifunctional conductive flexible stretchable hydrogels that show self-healing properties, adhesive abilities, and 3D printability. These hydrogels are made of nanoclay (laponite), multiwalled carbon nanotubes (CNTs), and N-isopropyl acrylamide. The hydrogels show quick response to heat and NIR light, along with good electrical conductivity, rapid self-healing, and flexible, stretchable mechanical properties. Additionally, these hydrogels demonstrate outstanding 3D printability, enabling printing into diverse shapes and 3D gridding scaffolds. These excellent properties of the hydrogels are demonstrated by the 3D bulky pressure-dependent device, human activity monitoring device, and 3D-printed gridding scaffolds (as shown in Figure 6IV) [38].

### 4.4. Biochemical Detection Sensors

Functional hydrogels exhibit electrical changes upon charge transfer caused by chemical reactions triggered by target analytes, which can be achieved by incorporating specific conductive fillers into the pristine hydrogel. Three-D printing techniques are particularly well-suited for fabricating next-generation hydrogel-based flexible chemical sensors, taking advantage of the ability to select from multiple additives and the inherent advantages of pre-programmed designs [37,107,123,129,130].

**Figure 7:**
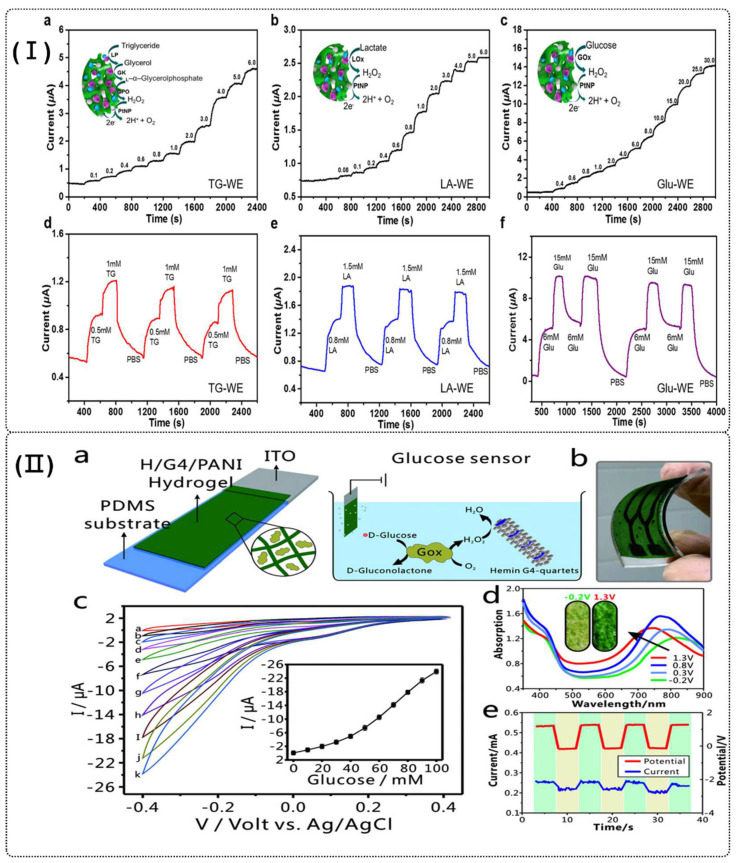
(**I**) Instant current–time response curves, repeatability, and sensing mechanisms of the corresponding metabolites of printed biosensors when metabolite solutions with different concentrations were pumped into the channel in an alternating manner (flow velocity: 200 μL/min). (**a**,**d**) TG-WE detects triglyceride. (**b**,**e**) LA-WE detects lactate. (**c**,**f**) Glu-WE detects glucose. The insets in (**a**–**c**) show the schematic sensing mechanisms of the corresponding metabolites by the PAni hydrogel/PtNP enzymatic biosensors [78]. (**II**) (**a**) Scheme of the glucose sensor based on GOx-loaded H/G4–PANI hydrogel for the detection of glucose. (**b**) The image of the glucose sensor. (**c**) Cyclic voltammograms of glucose sensor in the presence of different concentrations of glucose. Inset indicates the linear correlation of cathodic peak current with the concentration of glucose. (**d**) UV–Vis absorption spectra of the H/G4–PANI hydrogel film measured in HCl solution. Inset are the photographic images of the H/G4–PANI hydrogel film at different applied potentials. (**e**) Potential and chronoamperometry curves of the H/G4–PANI hydrogel film [130].

Li et al. reported on a “drop-on-demand” inkjet printing process for fabricating multiplexed biosensors using nanostructured conductive hydrogels. The process includes printing electrode material and different enzymes onto electrode arrays using a multi-nozzle inkjet system. The whole printing procedure is done in three rounds with just one alignment round, requiring about 5 min to create a page of sensor arrays with 96 working electrodes. These multiplexed assays can detect glucose, lactate, and triglycerides in real time with high selectivity and sensitivity (LOD = 0.07, 0.06, and 0.2 mM, respectively), yielding results comparable to those obtained in phosphate buffer solutions and calibration serum samples (as shown in Figure 7I) [78]. Zhong et al. developed an enzyme-like nanofibrous hydrogel by incorporating hemin into a G-quartet scaffold during cation-templated self-assembly with G and KB(OH)_4_. This hydrogel was further utilized to fabricate a semiconducting H/G4–PANI hydrogel for flexible electrochemical sensors. The co-assembly of cationic hemin with anionic borate esters enables a well-defined organization of catalytic active sites, leading to an efficient enzyme-like property in the hydrogel (as shown in Figure 7II). This nanofibrous semiconducting hydrogel with enzyme-like characteristics shows potential for creating artificial enzymes with novel functionalities and enhancing bioelectronic applications [130]. 

Further research could also explore the integration of these hydrogels with other emerging technologies, such as artificial intelligence (AI) and the Internet of Things (IoT). For example, the integration of AI algorithms with hydrogel-based sensors could lead to intelligent systems that predict and adapt to environmental changes, while IoT connectivity could facilitate remote monitoring and control of these systems. The development of these conductive and highly functional hydrogels for environmental sensing represents a significant advancement in materials science, with great potential across numerous industries. Even more innovative applications are expected, improving both everyday technologies and specialized industrial applications.

**Table 2:** Summary of representative 3D-printed conductive hydrogel-based sensors.

Hydrogels System and Component	Printing Techniques	Conductivity	Gauge Factor	Sensing Range	Application	Ref.
PPNGC hydrogel	N/A	2.1 S/m	0.78 (0–120% strain), 1.52 (120–600% strain)	0–600%	human motions	[111]
PANI hybrid hydrogel	DIW	N/A	2.2 (0–3% strain), 0.8 (40 ppm NH_3_), 7.3 (400 ppm NH_3_)	(1) stress sensing of 0–899.8 MPa, (2) strain sensing of 0–764.4%	human motions, NH_3_ detection, temperature detection	[108]
SMF/RSF/PAM composite hydrogel	DIW	0.056 S/m	0.9	0–200%	human motions	[112]
poly(ACMO)/pt hydrogel	DLP	1.6 S/cm	1.5 (0–10% strain), 7.2 (10–100% strain)	0–100%	human motions	[74]
polyaniline hybrid (PHH) hydrogels	DIW	0.04–0.21 S/m	∼1.77	0–820% (the resistive strain sensors)	human motions, human–machine interfaces, physiological signal detections	[32]
dorsal root ganglion (DRG) cell-encapsulated gelatin methacryloyl (GelMA) hydrogels	SLA	662.0~ 968.0 Ω/sq	N/A	N/A	healthcare applications, neural tissue regeneration	[85]
PPy/PEGDA hydrogel	DLP	2 MΩcm	N/A	N/A	biosensors, drug delivery, tissue engineering	[19]
PEGDA/AAm LiCl/nHAp hydrogels	projection microstereolithography (PμSL)	~0.1 S/cm	N/A	both large-scale and tiny human motions	human motions, conductor, neural interface	
PAINT hydrogels	DIW	0.26~0.58 S/m	signal-to-noise ratio by 88%	N/A	healthcare applications	[2]
GNPs–CNTs (GC) hydrogels	multi-jet fusion (MJF)	1.48 × 10^−2^ S/m	20.1(0–5% strain), 2.3 (6–26% strain), 360.8 (26–70% strain)	0–70% strain, 10~90% humidity	strain/humidity sensors	[115]
PEGDA/MWCNT-based composite hydrogel	multimaterial mask image projection-based stereolithography	770 kΩ~17.25 MΩ	R*rel* = 5.73 for 150 μm thickness to 2.17 for 450 μm thickness with 2 μL water	N/A	liquid sensing	[116]
PVA/agarose/ borax/liquid metal composite	three-dimensional printed molds	N/A	SNR = 4.05	N/A	multimodular sensor system biosignal detection	[33]
poly (N-isopropyl acrylamide)/polyaniline hydrogels	DIW	7.1 × 10^−6^ S/m	N/A	N/A	stimulus-response electronics, flexible electronics, artificial intelligence wearables	[124]
polyNCMA/LiTFSI gel	N/A	3.6 × 10^−5^ S/m at 30 °C, 2 × 10^−3^ S/m at 90 °C	8.4%/K	30~40 °C	temperature monitor	[117]
PEDOT:PSS/HPU gel	extrusion printing	N/A	N/A	pH 3~11	pH sensor	[123]
PEGDA–PANI electroconductive hydrogel	SLA	2.5 × 10^−2^ S/m		pH 2~7	smart biomedical sensors, pH sensor	[107]
(poly(ethylene glycol)-b-poly(propylene glycol)-b-poly(ethylene glycol) (PF127))/CNTs	N/A	0.16~0.20 S/m	N/A	N/A	wearable electronic devices, health monitoring	[38]
PANI-based biosensors	inkjet printing	N/A	(1) 7.4927 μA·mM^−1^·cm^−2^ of triglyceride, (2) 3.94 μA·mM^−1^·cm^−2^ of lactic acid, (3) 5.028 μA·mM^−1^·cm^−2^ of glucose	(1) 0.1–6 mM of triglyceride, (2) 0.5–1.7 mM of lactic acid, (3) 1–25 mM of glucose	biosensors for human health monitoring	[78]
GOx-loaded H/G4–PANI Hydrogel:	inkjet printing	N/A	N/A	H_2_O_2_ of (1–10) × 10^−3^ M	flexible bioelectronics	[130]

N/A indicates that this information is not applicable in the reference.

## 5. Conclusions and Perspectives

In summary, recent years have seen significant advancements in the fabrication, properties, and applications of 3D-printed conductive hydrogel sensors. Three-D printing offers unprecedented control over the design and fabrication of complex structures with high precision and customizability. By integrating conductive materials into hydrogel matrices, researchers have developed innovative strategies for 3D-printing conductive hydrogels with tailored properties and geometries. These sensors can be customized for specific applications, offering great potential for multifunctional smart sensors. 

This review begins by discussing representative strategies for fabricating conductive hydrogels and methods for enhancing their conductivity. It then highlights recently reported conductive hydrogels and details advancements achieved using different 3D printing techniques, each with its own strengths and limitations. Additionally, the review explores the diverse applications of 3D-printed conductive hydrogel sensors in various fields, such as human motion detection, strain sensing, temperature sensing, humidity sensing, and gas sensing. These applications demonstrate the versatility and potential of these materials in addressing real-world challenges in healthcare, environmental monitoring, human–machine interfaces, etc.

However, several challenges remain to integrate these 3D-printed conductive hydrogel sensors into daily life, requiring cooperation across chemistry, materials science, medicine, and engineering. For instance, it is still challenging to achieve a balance between mechanical and conductive properties. When integrating high-content conductive components into hydrogel matrices, it is difficult to avoid a decline in the mechanical properties of the hydrogel due to the incompatibility of the conductive components and the hydrophilic polymer network of the hydrogel. Continuous efforts are needed to develop new conductive hydrogel formulations with improved conductivity and mechanical properties. The incorporation of advanced conductive components (graphene, carbon nanotubes, and conductive polymers) as well as optimal network design, hold promise for improving the performance of these materials.

The long-term stability of hydrogel sensors is a significant concern. Once exposed to the environment, most hydrogel materials may lose water, resulting in unstable performance. Hydrogels with an appropriate water content offer excellent properties such as flexibility, tissue similarity, and good mechanical properties. However, dehydration causes hydrogels to harden, become brittle, and decline in sensitivity. Water loss can be mitigated through some methods, such as surface modification or solvent replacement, but these methods may lead to the decrease of the hydrogel’s mechanical or electronic and sensing properties. For practical applications, ensuring the long-term stability of flexible sensors is crucial and warrants further exploration and study.

In addition, some conductive hydrogels exhibit excellent biocompatibility, making them promising candidates for biomedical applications, including tissue engineering, drug delivery, and implantable devices. Further research is needed to explore their potential in these areas, addressing challenges related to long-term stability, biocompatibility, and integration with biological systems.

Most of the hydrogel sensors currently reported offer singular detection functions. Beyond stress and strain signals, variations in physiological parameters like temperature, humidity, and pH can effectively indicate health changes. Therefore, creating hydrogel biosensors with rapid response rates, heightened sensitivity, and broad detection ranges for temperature, humidity, and pH signals shows significant potential for smart wearable and implantable technologies.

Furthermore, while 3D printing offers flexibility and customization, scaling up the production of conductive hydrogel sensors for commercial applications remains a challenge. Ongoing efforts should focus on developing excellent sensing properties and high-precision, high-throughput, and cost-effective manufacturing processes that maintain desired properties and performance.

Overall, interdisciplinary collaboration between researchers in materials science, engineering, chemistry, and biology is important to address challenges and realize the full potential of 3D-printed conductive hydrogel sensors. With continued innovation and research, we believe that these advances of 3D-printed conductive hydrogel sensors will have a significant impact in transforming industries and improving our quality of life.

## Figures and Tables

**Table 1:** Summary of 3D printing technologies for conductive hydrogel fabrication.

3D Printing Technology	Material Requirements	Resolution	Characteristics	Limitations	Ref.
Inkjet Printing	Conductive inks (e.g., silver nanoparticle ink, graphene ink)	~20–50 μm	Precise material deposition, good for thin and complex layers	Lower mechanical strength, ink formulation critical	[78,79,80]
Direct Ink Writing (DIW)	Shear-thinning materials, conductive hydrogels	~100–300 μm	Customized viscosity, highly adaptable to various materials	Complex multi-step process, a temporary sacrificial material	[34,81,82]
Stereolithography (SLA)	Photopolymers doped with conductive materials	~50–200 μm	High resolution, complex structures achievable	Limited material choices, brittle structures	[85]
Digital Light Processing (DLP)	Photopolymers combined with conductive powders or fibers	~25–100 μm	High resolution, fast printing speed	Material restrictions, post-processing needed	[16,19,83,84]
Two-Photon Polymerization (TPP)	Photosensitive conductive hydrogels	<1 μm	Ultra-high resolution, capable of nanoscale features	Expensive equipment, limited scalability of photo-initiators	[86,87]

## Data Availability

Not applicable.
